# Challenges in comparing the quality of life of older people between ethnic groups, and the implications for national well-being indicators: a secondary analysis of two cross-sectional surveys

**DOI:** 10.1186/1477-7525-9-109

**Published:** 2011-12-05

**Authors:** Robert L Grant, Ann Bowling

**Affiliations:** 1 Faculty of Health and Social Care Sciences, Kingston University and St George's, University of London, UK

**Keywords:** Older people, ethnicity, quality of life, socio-economic status, well-being

## Abstract

**Background:**

The current international interest in well-being indicators among governmental agencies means that many quality of life scales are potential components of such national indicator sets. Measuring well-being in minority groups is complex and challenging. Scales are available that have been validated in specific parts of the population, such as older people. However, validation among combinations of minority groups, such as older adults of ethnic minority backgrounds, is lacking.

**Findings:**

We pooled data from two surveys of older adults in Great Britain: one conducted among White British people, and one among four ethnic minority groups. Quality of life was measured by the Older People's Quality of Life (OPQOL); Control, Autonomy, Self-realisation, Pleasure (CASP-19); and World Health Organization Quality of Life scale for older people (WHOQOL-OLD). We found differences, some significant, between groups in terms of self-reported importance of various aspects of quality of life. A regression model of each total quality of life scale revealed greater unexplained variability in the White British group than the others. Principal components analysis within each ethnic group's data showed considerable differences in the correlation structures.

**Conclusions:**

There are differences between ethnic groups that are consistent across the three scales and are not explained by a battery of predictor variables. If scales such as these are used to compare quality of life between ethnic groups, or equivalently between geographical regions, the different results in each group are liable to bias any comparison which could lead to inequitable policy decisions.

## Introduction

Policy makers world-wide are increasingly interested in promoting and measuring societal well-being, which is a dynamic, multi-faceted concept with social and psychological dimensions, overlapping with measures of quality of life (QOL) [1-3]. A great deal of the well-being of any nation will be determined by physical and mental health and existing QOL scales are being examined as potential well-being indicators. The aim of incorporating well-being measures into governmental policy is hopefully to stimulate improvements over time and to promote equity, as defined most recently by ongoing work in the United Kingdom and Europe [4,5]. The well-being of minority and marginalised groups, whose members are typically disadvantaged, will be key to success, but creating a set of indicators that is valid for such groups is a considerable challenge. Official statistics struggle to include accurate information on "hard to reach" groups, often relying on assumptions and extrapolation. Bajekal and colleagues reported differences between ethnic groups in seven facets of QOL among older people in a national survey in England and Wales [6]. It is unknown whether such variations are attributable to differences in priorities or true underlying QOL. Most measures have been developed largely by experts with some lay input, although more recently developed QOL scales have used a grounded approach incorporating participants' priorities [7].

### Aims

In this paper, we investigate the challenges in applying QOL scales to different ethnic groups. We have considered three QOL measures which are specifically validated for older people and sought to answer three questions:

• Do ethnic groups differ in the importance they allocate to different aspects of well-being?

• Are differences in self-reported importance associated with QOL, is this affected by individuals' characteristics, and does this differ between ethnic groups?

• Does the pattern of variability between individuals, measured by correlations among the QOL questions, differ between ethnic groups or age bands?

### Data sources

We analysed data from two surveys of community-dwelling older people in England, one among White British people and one among ethnic minorities. In each of these surveys, three quality of life measures were collected (OPQOL, CASP and WHOQOL-OLD [7]), along with covariates and subjective importance of seven aspects of well-being. The surveys were the ONS Omnibus (n = 555 white British participants over age 65, 16 from other ethnic groups were set aside), and Ethnibus (ethnic minority doorstep survey stratified by electoral ward on the basis of Census data: n = 152 Indian, 117 Pakistani, 86 Caribbean, 45 Chinese, all aged over 60) [8]. Both were based on home interviews with trained interviewers during 2007-8. For this reason, data were rarely missing: 931/955 had complete data for OPQOL, 942 for CASP and 905 for WHOQOL-OLD. Original data were compiled and processed in SPSS software; analyses for this paper were carried out in Stata version 11.

## Findings

### Self-reported importance of topics

**Figure 1:**
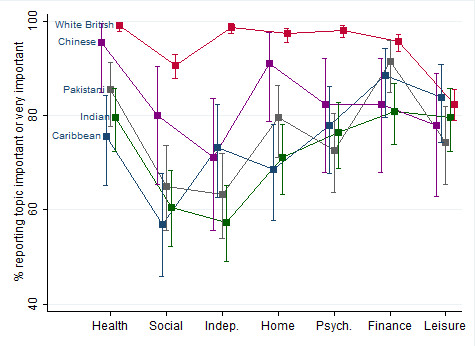
**Differences by ethnicity in perceived importance of different aspects of well-being (bars are 95% confidence intervals)**.

Participants' self-reported importance ratings for each of seven aspects of well-being (health, social, independence, home, psychological, financial, leisure) were collected by the categories "less important", "important" and "very important". We considered proportional odds models for ordinal data but found the proportionality assumption very unlikely based on graphs of cumulative log-odds, and so the ratings were dichotomised into less important vs. (important or very important). Using mixed-effects logistic regression (which adjusts for unexplained variation between individuals), adjusting for age (negative correlation for all topics) and household size (positive correlation for all topics), we found that ethnic groups differed in the reported importance [Figure 1]. The largest differences are between White British participants (almost all of whom rate all the topics as important) and the others, though the Chinese participants returned significantly higher importance in some topics. Adjusting for the covariates inflated ethnic differences, which is likely to be because ethnic minorities were on average younger and living in larger households.

### Association between importance and quality of life

Importance was compared with OPQOL sub-scale scores through rank correlations because the topics relate directly to the sub-scales. Greater importance of topics is correlated with higher OPQOL scores in the health (rho = 0.40), social (rho = 0.48), independence (rho = 0.60), home (rho = 0.46) and psychological (rho = 0.42) sub-scales. The others were weaker: financial (rho = 0.13) and leisure (rho = 0.24). Some of this correlation may be mediated by cultural differences or socio-economic status.

Linear regression models were constructed for each of the QOL total scores (OPQOL, CASP and WHOQOL-OLD), incorporating survey oversampling by inverse probability weighting (for each combination of ethnic group and region). Assumptions were examined graphically. An assumption about including mixed race British-Caribbean numbers from the 2001 UK Census within the sampling frame for "Caribbean" participants was tested by sensitivity analysis, and found not to affect results. Geographical region, age, ethnic group, housing tenure and some of the importance ratings were significant predictors of total QOL scores. Predictors were very similar for the three scales [Table 1], though the weaker effect of ethnicity on the CASP scores may arise because this scale was not tested in ethnic minority groups (in the British context) [7]. Where interactions are seen between ethnicity and another predictor, this is never solely attributable to White British vs. all others, so in none of the analyses can this mask a methodological difference between the Omnibus and Ethnibus surveys.

**Table 1:** Linear regression coefficients (Region and ethnicity had significant interactions predicting OPQOL and CASP.

		OPQOL		CASP-19		WHOQOL-OLD	
Predictor		Coefficient (95% CI)	p-value	Coefficient (95% CI)	p-value	Coefficient (95% CI)	p-value
Ethnic group:	White British (baseline)						
	Indian	-11.1 (-13.5, -8.7)	< 0.001	3.8 (2.2, 5.4)	< 0.001	-9.3 (-10.6, -8.1)	< 0.001
	Pakistani	-9.9 (-12.5, -7.3)	< 0.001	-0.1 (-1.8, 1.7)	0.95	-8.0 (-9.6, -6.4)	< 0.001
	Caribbean	-6.1 (-8.8, -3.4)	< 0.001	0.7 (-1.0, 2.5)	0.79	-9.4 (-10.5, -8.2)	< 0.001
	Chinese	-2.5 (-5.5, 0.6)	0.12	4.5 (2.0, 6.9)	< 0.001	-3.2 (-4.9, -1.4)	< 0.001

Region:	London (baseline)						
	Midlands	4.7 (2.6, 6.8)	< 0.001	2.1 (1.0, 3.3)	< 0.001	Not significant	
	South	5.5 (3.4, 7.7)	< 0.001	3.2 (2.0, 4.3)	< 0.001		
	North	2.8 (0.7, 4.8)	0.01	1.6 (0.4, 2.7)	0.01		
	Wales/Scotland	3.5 (1.3, 5.8)	0.002	2.4 (1.1, 3.7)	< 0.001		

Social aspects:	"very important" (baseline)						
	"important"	-3.5 (-4.5, -2.5)	< 0.001	-2.1 (-2.7, -1.6)	< 0.001	-4.9 (-5.7, -4.0)	< 0.001
	"not important"	-8.6 (-10.0, -7.2)	< 0.001	-5.0 (-6.1, -4.0)	< 0.001	-10.3 (-11.7, -8.9)	< 0.001

Home aspects:	"very important" (baseline)						
	"important"	-2.7 (-3.6, -1.8)	< 0.001	-0.3 (-0.9, 0.2)	0.25	-1.6 (-2.3, -0.8)	< 0.001
	"not important"	-10.9 (-12.6, -9.1)	< 0.001	-4.1 (-5.2, -3.0)	< 0.001	-4.7 (-6.0, -3.4)	< 0.001

Psychological aspects:	"very important" (baseline)						
	"important"	-2.3 (-3.1, -1.5)	< 0.001	-0.6 (-1.1, -0.1)	0.02	Not significant	
	"not important"	-5.4 (-6.5, -4.4)	< 0.001	-2.2 (-3.0, -1.4)	< 0.001		

Leisure aspects:	"very important" (baseline)						
	"important"	-3.8 (-4.7, -2.8)	< 0.001	-2.0 (-2.5, -1.4)	< 0.001	-0.2 (-1.0, 0.6)	0.57
	"not important"	-9.2 (-10.6, -7.8)	< 0.001	-6.9 (-7.8, -6.1)	< 0.001	-4.4 (-5.7, -3.2)	< 0.001

Tenancy:	Owned (baseline)						
	Rented	-6.3 (-7.2, -5.5)	< 0.001	-2.1 (-2.6, -1.5)	< 0.001	-5.4 (-6.3, -4.6)	< 0.001
	Free	-2.9 (-4.7, -1.1)	0.002	-1.9 (-3.0, -0.9)	< 0.001	-4.1 (-5.3, -3.0)	< 0.001

Age (years):		Not significant		-0.08 (-0.11, -0.04)	< 0.001	Not significant	

Importance of leisure aspects and ethnicity had significant interactions predicting OPQOL and CASP. Importance of social aspects and ethnicity had significant interactions predicting CASP and WHOQOL-OLD.)

Gender, first language, marital status and the number of adults living in the household were also considered but were not significant predictors of any of the QOL scales. Socio-economic status cannot be included fully in the regression because there are different measures in the two surveys, but we have used housing tenure as a proxy. Rank correlations within each survey's data suggest that socio-economic status is weakly positively correlated with all three measures in Omnibus, but not associated with any of the measures in Ethnibus. Whether this is due to ethnicity or an artefact of the particular social class measure is debatable.

**Figure 2:**
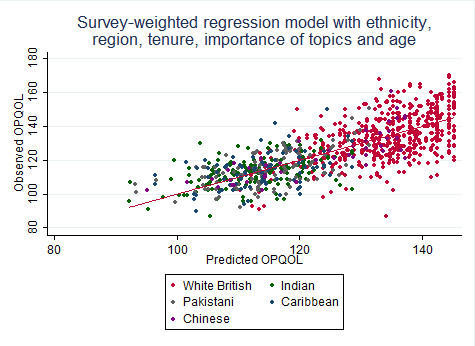
**The best statistical model does not explain enough of the variability in the white British participants**.

Quality of life differs markedly between ethnic groups, even after adjusting for the predictors listed above. The standard deviations of the QOL scores differ between ethnic groups, and the models do not account for all of this, leading to heteroscedasticity in all three models (unexplained variation increases with higher predicted quality of life). This could arise because of the relative homogeneity of the individuals' characteristics in the white British group. This suggests that there are differences between the ethnic groups that we cannot fully explain. [Figure 2**] **The weak correlation with socio-economic status suggests that obtaining a better measure will not solve the problem alone.

### Patterns of variability within each ethnic group and age band

**Figure 3:**
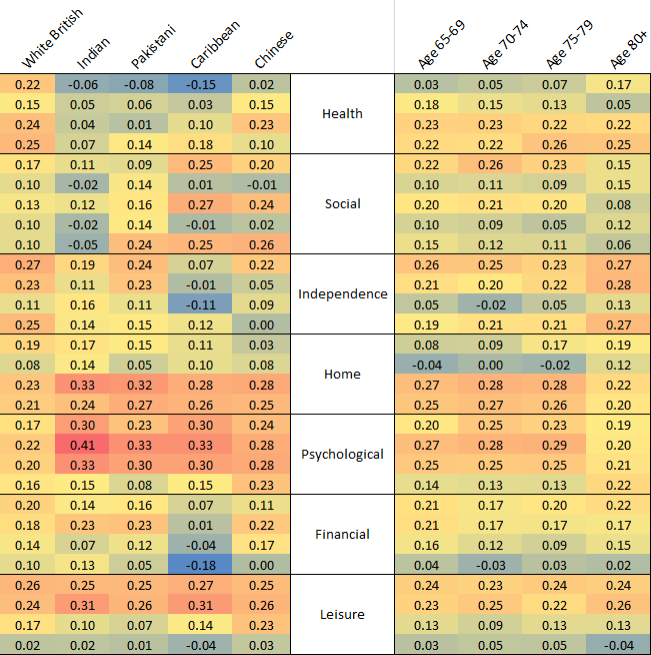
**Weights from ethnicity-specific principal components analyses differ notably as seen in this heatmap; age-specific differences are smaller**. High weights (at the red end of the spectrum) indicate OPQOL questions on which individuals differ more, and hence the questions contribute strongly to an optimum summary score for that group.

Principal components analyses were conducted on individual OPQOL questions for different ethnic groups and in 5-year age bands, and the resulting ideal ethnicity-specific summary weights were compared [9]. Differences in these weights suggest that individuals in different groups differ in subtle ways that cannot be fully captured in a single composite measure [Figure 3].

## Conclusions

We have seen that our data do not provide simple solutions to the problem of making fair comparisons between ethnic groups. Regional comparisons will similarly be affected because of differing population characteristics. There are two main limitations. Firstly, we cannot fully adjust for socio-economic status, which is known to be difficult to measure in retired people [10]. We have used housing tenure as a proxy because of its availability in the data but we recognise that it is a coarse indicator. Secondly, we cannot rule out differences between ethnic groups arising from differences between the two surveys. There were only 16 people in Omnibus who did not regard themselves as White British, not enough to allow us to test this by comparing them with Ethnibus. The Ethnibus participants were younger on average than the Omnibus, but we have adjusted for age in our analyses.

The differences seen between ethnic groups could as plausibly arise from cultural norms, expectations or semantics, as from community structures and economic disadvantage, but regardless of the reason, the fact that inter-group differences can be distorted is enough to raise concern. The WHOQOL-OLD scale was developed by modifying a generic QOL scale for adults and tested by a series of convenience samples internationally, which may explain its detection of fewer differences between ethnic groups compared to OPQOL. Before any QOL scale can be used as a well-being measure, it would need to be developed or tested in a variety of socio-cultural groups in the population, not simply analysed by groups to assess differences. The potential exists for policy informed by well-being statistics inadvertently to increase inequity or assign resources inefficiently because of over-simplification. As we have seen problems in comparing ethnic minorities' well-being, we can also expect problems in other minority and marginalised groups such as older people, children, recent immigrants, mental health service users, those whose first language is not English, those living in institutional care, homeless people and disabled people including communication difficulties.

## List of abbreviations

CASP-19: Control, Autonomy, Satisfaction, Pleasure scale; OPQOL: Older People's Quality of Life scale; QOL: quality of life; WHOQOL-OLD: World Health Organization Quality of Life scale for older people.

## Competing interests

The authors declare that they have no competing interests.

## Authors' contributions

Both authors contributed to writing, RG carried out secondary data analysis and AB was responsible for data collection and processing. Both authors have read and approved the final manuscript
